# Relationship between diagnostic accuracy and self-confidence among medical students when using Google search: A mixed-method study

**DOI:** 10.1371/journal.pone.0332918

**Published:** 2025-09-19

**Authors:** Yasutaka Yanagita, Kosuke Ishizuka, Daiki Yokokawa, Kiyoshi Shikino

**Affiliations:** 1 Department of General Medicine, Chiba University Hospital, Chiba, Japan; 2 Department of General Medicine, Yokohama City University School of Medicine, Kanagawa, Japan; 3 Department of Community-Oriented Medical Education, Chiba University Graduate School of Medicine, Chiba, Japan; Covenant University, NIGERIA

## Abstract

**Background:**

With the growing volume of medical information, proficiency in utilizing clinical decision support systems (CDSSs) is increasingly important for physicians. Further, research has primarily focused on CDSSs’ accuracy for specific symptoms, diseases, and treatments, but the extent to which CDSSs contribute to the clinical reasoning process and evaluation of their output remains unclear. While Google is not a traditional CDSS, previous studies have evaluated its role as a diagnostic support tool, demonstrating its ability to assist physicians in retrieving relevant medical information and influencing diagnostic decision-making.

**Objective:**

This study aimed to assess whether using Google search can enhance diagnostic accuracy and confidence among medical students, and to evaluate how the interpretation of search results influences their diagnostic confidence.

**Methods:**

Forty-eight fifth-year medical students in clinical clerkship at Chiba University Hospital were presented with ten clinical scenarios in text format. Initially, they provided the most likely diagnosis without assistance and recorded their confidence levels. Subsequently, they used Google search to revisit their diagnoses and confidence levels, using a 7-point Likert Scale. Focus group interviews were conducted to discuss changes in confidence, and the interviews were analyzed qualitatively using content analysis. A mixed-methods analysis compared the average number of correct diagnoses and confidence levels before and after using Google search.

**Results:**

In total, 470 responses from 48 fifth-year medical students were evaluated after excluding 10 inappropriate responses. Correct diagnoses increased from an average of 63.6% without assistance to 76.2% using Google search (*P* < .001), and confidence levels rose from 4.9 to 5.9 (*P* < .001). Qualitative analysis of higher-confidence responses identified 108 codes within 17 subcategories related to diagnostic processes.

**Conclusions:**

This study underscores the value of using Google search in medical education to enhance diagnostic skills and confidence. The improvement in accuracy and confidence among students demonstrates the supportive role of Google search in clinical reasoning and education. This highlights the need for educators to teach discernment in information analysis to ensure optimal use of CDSS in medical training. Proper integration of these tools is crucial for developing future physicians capable of effectively navigating vast amounts of medical data.

## Introduction

With advancements in medical information technology, physicians must rapidly gather useful information for diagnosis and treatment using various tools. Clinical decision support systems (CDSSs) provide inferred diseases and evidence-based information to medical practitioners based on guidelines and research [1]. CDSSs are recognized for their contribution to improving diagnostic accuracy and reducing diagnostic errors [2]. By entering patient complaints into a CDSS, physicians can recall related diseases and differential diagnoses and identify often overlooked diseases in challenging cases. This is particularly beneficial for initiating physicians who may struggle to recall appropriate differential diagnoses, making CDSSs especially valuable in primary care [3]. CDSSs can also prevent diagnostic errors due to bias in experienced users [4]. CDSSs are designed to function within structured clinical frameworks, relying on validated medical databases and predefined algorithms to generate recommendations. In contrast, search engines, such as Google, retrieve information from an extensive range of web-based sources, including both reliable and unverified content, and are highly dependent on user input. Given these fundamental differences, search engines cannot be classified as traditional CDSSs. However, they serve as accessible external resources that may assist in clinical reasoning by providing relevant medical information. The usefulness of Google as a web-based diagnostic support tool has been studied [5]. As the volume of medical information increases, the effective use of both traditional CDSSs and other digital information retrieval tools, such as search engines, becomes more critical for diagnosing and treating atypical and rare diseases that cause multiple symptoms [6]. Google search is not a typical CDSS that shows differential disease lists and treatment options; however, it is easily accessible to many health care providers, allowing them to quickly search and find medical information. Although it does not provide structured clinical decision support, Google search may still influence diagnostic reasoning by offering diverse sources of information that physicians can evaluate and integrate into their decision-making process. We hypothesized that the use of Google search, an easily accessible and expedient platform, would also improve diagnostic accuracy and reliability. We also expected that the resulting questionnaires and interviews would highlight the importance of critically evaluating Google’s output, clarifying the essential aspects of how search engines can be used as supplementary tools in clinical decision-making rather than as conventional CDSSs.

The ability to use digital health-related systems is essential for future medical practitioners [7,8]. It is important to start using such Information and Communication Technology systems as medical students. The Japanese model core curriculum for medical education now includes “the ability to use information science and technology” as a required competency, emphasizing the need to acquire this skill during pre-graduate education [9]. To maximize the effectiveness of a CDSS, it is crucial to understand its characteristics and use appropriate keywords, known as semantic qualifiers (SQ) for input [10,11]. Equally important is the ability to evaluate the output content. In addition, users should be careful not to rely too heavily on the system when making decisions. Regarding confidence and using the system to identify a diagnosis—increased confidence promotes assertiveness in clinical decision-making; however, a false sense of confidence can lead to clinical errors, especially if the diagnosis is incorrect. Thus, it is essential to strike a balance between confidence and critical evaluation of the system’s output.

While research has primarily focused on the accuracy of CDSSs for specific symptoms, diseases, and treatments [12], the extent to which search engines, as an alternative digital tool, contribute to the clinical reasoning process and to the evaluation of their output remains unclear. Clinical reasoning is a fundamental skill for physicians, involving the process of gathering, analyzing, and synthesizing clinical information to reach a diagnosis. Despite increasing reliance on digital tools, the way in which search engines influence the development of clinical reasoning skills has not been fully explored. Many CDSSs are expensive, making Google a free alternative for information retrieval [13].

This study aimed to evaluate whether using Google search free search engine by medical students improved their diagnostic accuracy and confidence. Furthermore, this study aimed to examine how Google search use impacts clinical reasoning by analyzing changes in diagnostic approaches and evaluating the importance of assessing output content for effective CDSS use.

## Methods

### Study design overview

**Fig 1:**
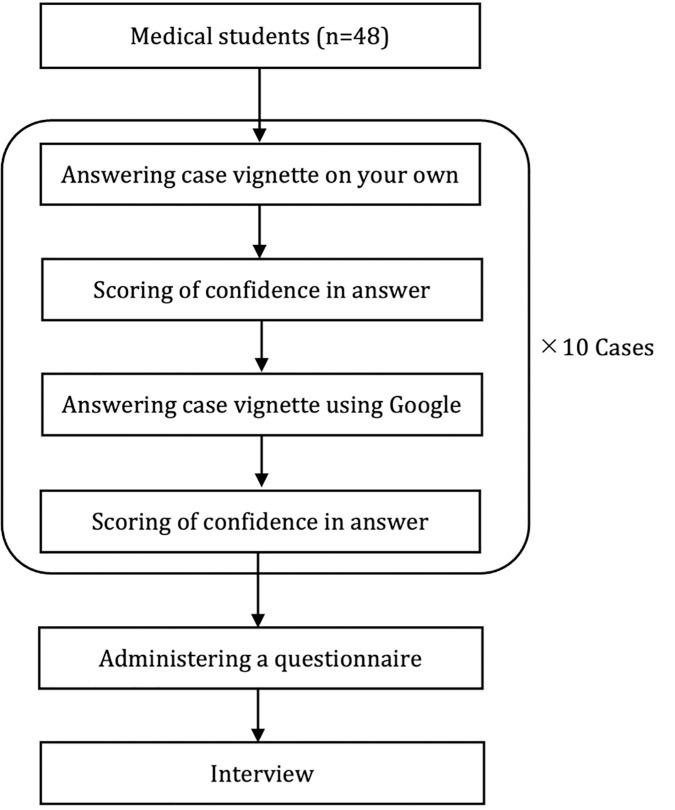
Flowchart of participants’ answers to questions and survey responses.

This study employed a mixed-method design with a pragmatic approach, combining quantitative and qualitative methodologies [14–17]. This design maximizes the strengths of both methodologies while minimizing their weaknesses, providing a deeper understanding of the results and incorporating participants’ perspectives. As quantitative research, a 10-question paper-based short description of a patient’s medical case used for educational purposes (clinical vignette) was presented online to medical students participating in a clinical clerkship via Microsoft Forms (Microsoft, US). For each question, participants were first asked to provide the most likely diagnosis without using any diagnostic support systems or textbooks and to indicate their confidence level in the diagnosis. Next, they answered the same question again, this time using a Google search, and recorded the diagnosis and confidence level quantitatively. The response flow consisted of ten questions. After completing all the questions, participants immediately filled out a questionnaire. At that time, focus group interviews were conducted on the reasons for the change in confidence. The results will be embedded as qualitative data to understand the contribution of Google search to diagnostic confidence (Fig 1). The evaluation assessed the usefulness of Google search by comparing the average number of correct answers and the average confidence levels before and after using Google search. The reasons for changes in diagnostic confidence were explored through the interview results. To improve the study’s quality, quantitative data were analyzed based on the CONSORT statement [18], and qualitative data were analyzed using the consolidated criteria for reporting qualitative research checklist [19].

### Participants and context

Forty-eight fifth-year medical students in a clinical clerkship at Chiba University Hospital participated in the study from 9^th^ March to 27^th^ July 2022. Medical schools in Japan are six-year institutions [20]. Participation was voluntary. Participants who were informed about the study and gave their consent were included, while those who did not give their consent were excluded. Furthermore, of the case vignettes answered by each participant, we excluded those with incomplete responses or those who answered with more than one diagnosis (only those with a single diagnosis in their response were included). For the questionnaires, those with incomplete answers were excluded, and the rest were included. Before participating, students demonstrated a certain level of competence by passing exams that assessed knowledge, problem-solving skills, and the Objective Structured Clinical Examination, which evaluates attitudes and examination skills. These examinations are administered nationwide to ensure the quality of students. Therefore, participants had learned the basic approach to clinical reasoning and understood the concept of SQ as search keywords.

### Experiment materials

To develop the case vignettes, focus group discussions were held with two faculty advisors (YY and KS) from the Department of General Medicine. The vignettes included only age, sex, chief complaint, and medical history, excluding physical examination or laboratory findings (S1 Samples). The vignettes focused on medical history to emphasize the initial stage of the diagnostic process, where medical history plays an important role in confirming the differential diagnosis, based on previous research [21]. The difficulty level was based on the list of diseases encountered during clinical training by the Ministry of Health, Labor and Welfare [22] and the National Medical Examination [23]. Twenty case vignettes on high-frequency diseases were created, assuming a correct response rate of over 60%, based on the passing score of the Japanese national medical exam. A cognitive debriefing on the case questions was conducted. Given that a correlation coefficient of 0.1–0.3 between cases indicates case specificity [24] and that ten questions are sufficient to achieve a reliability coefficient of 0.7 or more for case specificity, assuming a disease coefficient of 0.2 [25], we selected ten questions that were answered correctly by all physicians. Finally, the ten vignettes answered 100% correctly by seven general practice physicians in their third to seventh year of medical practice were included (S1 Lists). Participants were asked to respond to a questionnaire regarding the difficulty level of the vignettes and their proficiency level of Google on a 7-point Likert scale. For the difficulty level of the vignettes, participants provided a score between 1 (easy) and 7 (difficult) in response to the question, “How difficult is the case problem?” For Google proficiency, they provided a score between 1 (unfamiliar) and 7 (familiar) in response to the question, “Are you familiar with using Google as your usual tool for searching for medical information?”.

### Procedure

Before answering the case vignettes, participants received a demonstration on how to search Google, ensuring their response methods and skills in using Google were standardized. During the demonstration, participants accessed the Google home page, entered one to several specific symptoms as search terms, and pressed the search button. Then, from the search results displayed, they clicked on the information they wished to examine. After a single search was completed, the participant returned to the top page at the start of the search. No directives were given on what keywords to enter or what information to look for. Two faculty members (YY and KS) participated in the study, with one faculty member conducting the demonstration. Participants were informed that their grades would not be affected by their percentage of correct answers in this study, and they agreed not to share the case vignettes with others.

### Quantitative data

Case vignettes were presented using Microsoft Forms (Microsoft Corp., Redmond, WA, USA). The URL for each case vignette was provided, and participants accessed and answered each vignette using their own devices. Initially, participants were asked to provide a diagnosis for each case. They were then asked to respond on a 7-point Likert scale to the question, “How confident are you of the diagnosis you answered? (very low: 1 point to very high: 7 points),” to record their confidence. A 7-point scale was chosen to allow for finer granularity in assessing confidence. The adoption of the 7-point Likert scale expands the range of response options, thereby enhancing our ability to discern nuanced variations in confidence [26]. Subsequently, they were asked to diagnose the same case using Google search and to respond to the confidence level of that diagnosis in the same way. A score of one was given for a correct diagnosis and zero for an incorrect diagnosis. The primary outcomes measured were the change in the number of correct answers and the mean change in the 7-point confidence level for each diagnosis before and after using Google search. After the ten case vignettes were answered, an online questionnaire was administered using Microsoft Forms regarding the difficulty of the vignettes and Google’s proficiency level.

#### Data analysis.

The required sample size for a two-tailed test of the difference in means between two groups was 34, assuming a significance level of 0.05, a power of 0.8, and an effect size of 0.5. In total, 48 medical students were included in the study, which was quantitatively sufficient, assuming some participants might drop out. All 48 participants were included in the qualitative sampling. Focus group interviews continued until theoretical saturation was achieved; otherwise, additional focus group interviews were conducted. All statistical analyses regarding the 7-point Likert scale of diagnostic confidence and the average number of correct answers were performed using SPSS Statistics for Windows 26.0 (IBM Corp., Armonk, NY, USA). Paired-sample t-tests were applied for pre- and post-Google comparisons when the data were normally distributed. When the assumption of normality could not be met, the Wilcoxon signed-rank test—a non-parametric alternative for paired data—was employed.

### Qualitative data

#### Semi-structured focus group interviews.

The interviewers for collecting qualitative data were two men, YY and KS, both supervisors in the Department of General Medicine; they led this study and held PhDs in medicine. They each provided medical education to students at a university hospital. Relationships with participants who responded to the questionnaire were established in advance using Google Skills. Participants were informed about the study’s purpose and the interviewers’ previous research. They agreed to participate voluntarily. Content analysis was employed to complement the quantitative data. Following questionnaire completion, the principal investigator (YY) and research collaborator (KS) conducted focus group interviews using semi-structured questions (S1 Table). The participants were asked, “What change did you experience in your confidence level after using the tool, and why?” The interviewer recorded and transcribed the conversations with participants’ consent. The participants were not shown the faculty members’ personal opinions and behaviors. There were no repeat questionnaires, and participants’ transcripts were not reviewed, and no feedback requests were received. Focus group interviews were conducted with one group of two to four participants, depending on the number of participants rotating through our department. Focus group interviews were discontinued when the interview content from the participants reached theoretical saturation. Theoretical saturation involved collecting data, analyzing and coding it until the interviewers believed the data was saturated, then collecting additional data for confirmation, reanalysis, and verification that it was saturated [27].

#### Content analysis.

Content analysis was used to analyze the response categories in the qualitative research (Table 1) [16,17,28–30]. The collected responses underwent review where names and other identifying information were removed from the questionnaire, and statements were tabulated. To ensure study quality, an inductive approach was first applied, where two researchers (YY and KI) independently developed coding statements to categorize quotes from the focus group interviews based on changes in confidence levels. After coding, similar codes were grouped into subcategories. In the next stage, a deductive approach was used to further organize these subcategories according to the established framework of Working Definitions for the Different Components [31]. Components of Clinical Reasoning comprises a clinical reasoning process that includes Information gathering, Hypothesis generation, Problem representation, Differential diagnosis, Leading or working diagnosis, Diagnostic justification, and Management and treatment (S2 Table). To ensure the reliability of results, regular discussions and reviews were conducted. As for the validation process, measures were taken to ensure the reliability and validity of the coding. First, two evaluators independently coded subsets of the data, then compared the codes, and any differences were discussed and resolved multiple times. We also shared a portion of the post-discussion coding with the third researcher, KS, who has experience in qualitative research, to confirm that the evaluators’ interpretations were accurate.

**Table 1:** The step of qualitative content analysis.

Diagnostic Confidence	
Step	**Description**
Step 1: Overview	The collected responses underwent review where names and other identifying information were removed, and comments were tabulated. A coding system was developed. 1. Open coding: Review some of the comments obtained from the focus group interviews. Labeling of increased or decreased confidence and coding of content was done. 2. Axial coding: All sample of comments were reviewed. Specific passages belonging under subcategories identified in initial open coding were tagged. 3. Selective coding: The researchers went over each document, looking for passages that were incorrectly categorized and contradicting information.
Step 2: Independent analysis	Two researchers (YY, KI) independently read all open-ended comments and performed the initial coding. They compiled labels, subcategories for analysis. Similar codes were then grouped together as subcategories, and the subcategories were then categorized according to the seven Components of Clinical Reasoning.
Step 3: Discussion of subcategory	To ensure the quality of the study, researcher triangulation was conducted by having two researchers (YY, KI) discuss, identify, and agree on the coding of descriptors.
Step 4: Interpretation and verification	After classification, regular discussions and reviews were conducted by researchers, including KS (experienced qualitative researchers), to ensure the reliability of the findings and the interpretation of the meanings derived from the survey. The Consolidated Criteria for Reporting Qualitative Research (COREQ) checklist was used to report survey results.
Step 5: Comparison and theory	The research findings were compared with relevant literature and theories. Implications for future research and reforms were outlined.

### Ethics approvals

This study received approval from the Graduate School of Medicine Ethics Review Committee of Chiba University, Chiba, Japan. Procedures for obtaining informed consent were explained to participants, and consent was obtained orally after ensuring sufficient understanding. This study was registered in the University Hospital Medical Information Network Clinical Trials Registry (UMIN-CRT) (UMIN 000048613).

## Results

### Participant characteristics and diagnostic accuracy and confidence changes

Participants had a median age of 23 years (range: 22–24). The percentage of male participants was 89.5% (Table 2).

**Table 2:** Participant characteristics.

Characteristics	
Age median years, years (range)	23 (22 –24 )
Sex male, n (%)	43 (89.5)

**Fig 2:**
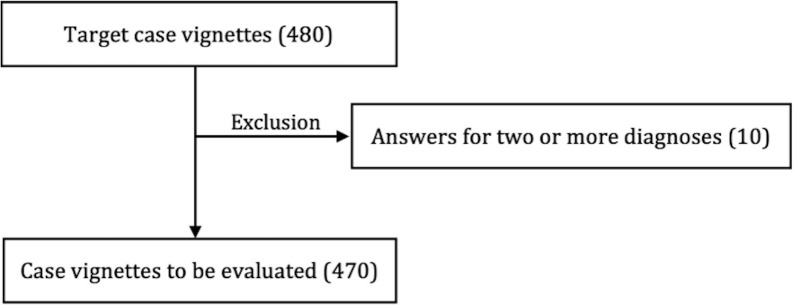
Case vignettes to be evaluated.

Out of 480 targeted questions, 10 were excluded owing to multiple answers (Fig 2). In total, 470 questions (97.9%) were analyzed, with correct diagnoses accounting for 63.6% (299/470) without using Google search, and 76.2% (358/470) with Google search. The median number of correct answers per participant was 7.0 without using Google search and 8.0 with Google search. The difference was statistically significant according to the Wilcoxon signed-rank test (*Z* = 4.6, *P* < .001, *r* = 0.66), indicating improved diagnostic accuracy when using Google search. Diagnostic confidence also increased significantly, rising from 4.9 to 5.9 on a 7-point scale, as determined by the Wilcoxon signed-rank test (*Z* = 6.0, *P* < .001, *r* = 0.87) (Table 3). Table 4 shows the change in confidence across four categories of correct and incorrect answers, with and without Google use, for the 470 questions. When a correct answer was given without Google and then a correct answer was given using Google, confidence increased by an average of 0.87 points. In addition, when an incorrect answer was given both without and with Google, confidence increased by an average of 1.2 points.

**Table 3:** Comparison of correct answers and diagnostic confidence with and without Google search.

	Without Google	With Google	*P* value
Correct answers, median	7.0	8.0	<.0.01*
Diagnostic Confidence, median	4.9	5.9	<.0.01*

* Wilcoxon signed-rank test.

**Table 4:** Change in diagnostic confidence with and without Google.

Without Google	With Google	Confidence				Average confidence changes points, n
		Increase, n		No change, n	Decrease, n	Total, n
Correct	**Correct**	176	100	10	286	0.87
Correct	**Incorrect**	8	3	2	13	0.46
Incorrect	**Correct**	64	6	2	72	2.00
Incorrect	**Incorrect**	75	19	5	99	1.20
Total		323	128	19	470	

### Questionnaire results: Case vignettes and Google proficiency

**Fig 3:**
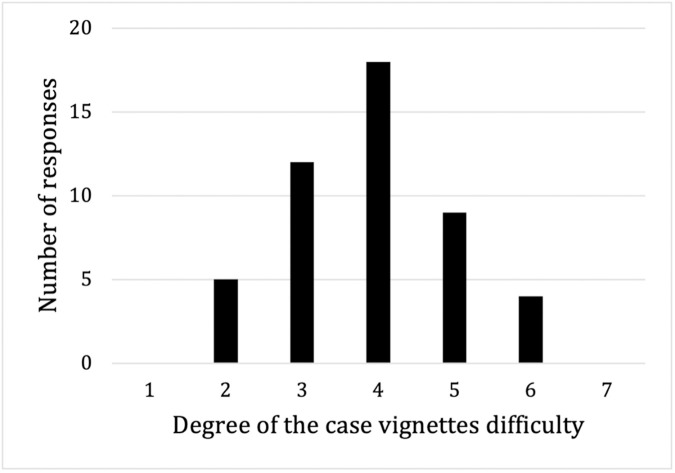
Difficulty of the case vignettes.

**Fig 4:**
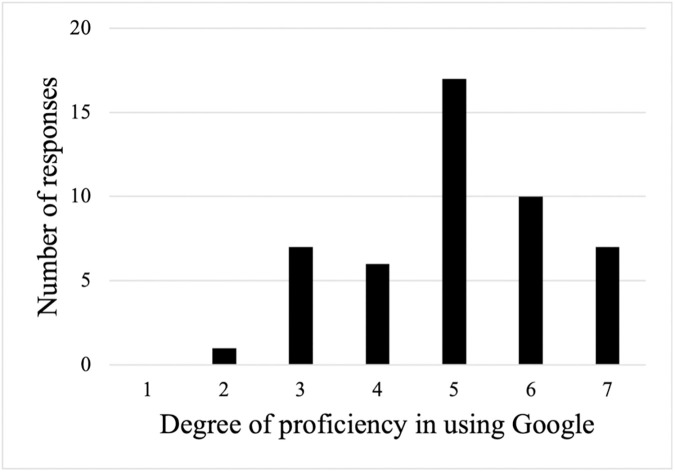
Proficiency in using Google.

After answering ten case vignettes, respondents were asked to rate the difficulty of the vignettes and their proficiency in using Google search on a 7-point scale. For vignette difficulty, a rating of 1 indicated “easy” and 7 indicated “difficult.” The results showed that 35.4% (17/48) of respondents rated the vignettes as “easy,” 37.5% (18/48) rated them as “neither easy nor difficult,” and 27.1% (13/48) rated them as “difficult” (Fig 3). The average percentage of correct answers according to the difficulty level that the participants responded to is presented in Table 5. Regarding proficiency in using Google, 70.1% (34/48) of respondents reported being proficient (Fig 4).

**Table 5:** Average of correct answers per difficulty level answered by participants.

Difficulty of cases	Number of Respondents, n	Average of correct answers without using Google, %
**1**	0	NA
**2**	5	84.0
**3**	12	61.2
**4**	18	62.4
**5**	9	64.2
**6**	4	49.7
**7**	0	NA
**Total**	48	63.6

* Difficulty level: 1 for easy, 7 for difficult.

### Content analysis

The focus group interviews were closed with 24 people because it was determined that theoretical saturation had been reached. A total of 108 codes were extracted from the interview content based on whether the group’s confidence level increased or decreased when responding to the case vignettes using Google. From these codes, 17 subcategories were generated. All seven components of clinical reasoning covered each subcategory. Eight subcategories were common to each of the groups with increased and decreased confidence. Five subcategories applied only to the group with increased confidence, and four subcategories applied only to the group with decreased confidence.

Tables 6 and 7 list the categories, subcategories, numbers of codes, and representative quotes. For the group with increasing confidence, the “Problem representation” category included subcategories like “Importance of input content” (eight codes) and “Selecting and choosing output information” (seven codes), totaling 18 codes. The “Hypothesis generation” category primarily involved “Disease recall assistance” (12 codes). In the group with lower confidence, the “Diagnostic justification” category included “Confusion due to output information” (11 codes), and the “Problem representation” category featured “Importance of input content” (three codes), totaling eight codes.

**Table 6:** Content analysis of groups with increased confidence.

Category		Subcategory		Quotes
Information gathering	(10)	Assist in the knowledge of diseases	(7)	*CDSS can compensate for insufficient medical knowledge.*
		Insufficient knowledge of the disease	(3)	*Using tools can be helpful for those who do not have the knowledge.*
Hypothesis generation	(16)	Disease recall assistance	(12)	*Using the CDSS, I can see that there are other diseases that I had not thought of.*
		Cognitive bias avoidance	(4)	*The use of CDSS is affected by anchoring vises.*
Problem representation	(18)	Importance of input content	(8)	*Anyway, I switched all the SQs to simple words and searched.*
		Selecting and choosing output information	(7)	*Websites of papers and diagnostic summaries gain more credibility.*
		Evaluation of output information	(3)	*Searching while assuming a particular disease will narrow down how information is obtained from the search results.*
Differential diagnosis	(10)	Review of differential diagnosis	(8)	*Differential diseases of headache and other symptoms were presented, and I chose the one that matched the symptoms of the case.*
		Verification of differential disease	(2)	*Symptoms can identify similar diseases.*
Leading or working diagnosis	(7)	How to use CDS	(7)	*I think it depends on the ability of the person using the CDSS and how they use it.*
Diagnostic justification	(13)	Validation of diagnosis	(12)	*I was convinced that if there was a diagnosis that I had thought of from the beginning and it matched the results of my research, then it was the correct diagnosis.*
		Assistance in narrowing down the disease	(1)	*CDSS could be used to narrow the differential diagnostic list for unfamiliar diseases.*
Management and treatment	(1)	Coping with common diseases	(1)	*Google is good for searching for common diseases, because common diseases are displayed first.*

*() number of code.

**Table 7:** Content analysis of groups with decreased confidence.

Category		Subcategory		Quotes
Information gathering	(4)	Information bias	(3)	*Common diseases, the most searched content in Google, come to the top of the list.*
		Insufficient knowledge of the disease	(1)	*Misdiagnosis increases when there is no knowledge of diseases.*
Hypothesis generation	(4)	Disease recall assistance	(3)	*If a differential diagnosis does not come to mind, the degree of certainty does not increase because it is impossible to choose a candidate from those that have been researched and found.*
		Cognitive bias avoidance	(1)	*Diagnosis may be made intuitively.*
Problem representation	(8)	Importance of input content	(3)	*It may be difficult to put in a proper SQ or the common diseases will be on top. The search results are affected by the words we input.*
		Confusion due to cognitive bias	(2)	*It depends on how you use the CDSS. Discarding information and bias can lead to more misdiagnoses.*
		Evaluation of output information	(2)	*Information on specific symptoms may lead to a diagnosis, but there may be no specific symptoms. This makes diagnosis difficult.*
		Cognitive bias	(1)	*The obsession with common diseases makes it impossible to consider rare diseases and increases diagnostic errors.*
Differential diagnosis	(5)	Verification of differential disease	(3)	*If I do not know the disease, I may assume the wrong disease is the correct one.*
		Review of differential diagnosis	(2)	*A Google search gave me many differential diagnoses.*
Leading or working diagnosis	(1)	How to use CDS	(1)	*Even medical professionals misdiagnose when they fail to use the CDSS properly.*
Diagnostic justification	(11)	Confusion due to output information	(11)	*Google outputs so much information that I answered questions without being sure.*

*() number of code.

## Discussion

In this study, we evaluated whether using Google search as a supplementary diagnostic tool improved medical students’ diagnostic accuracy and confidence. The results suggest that medical students achieve higher diagnostic accuracy when using Google search. Additionally, when students could recall a condition before using Google search, they focused on information supporting their diagnosis, increasing their confidence. Even when they could not recall the disease, they integrated their previous medical knowledge with the information provided by Google search, thus boosting their confidence. However, even if the diagnosis was incorrect, using Google the CDSS still increased their confidence level. These findings indicate that medical students using Google search need to have a clear focus on the output information and the ability to accurately evaluate it. In another study examining diagnostic accuracy using Google search, medical students’ diagnostic accuracy significantly improved. The diagnostic accuracy observed in this study aligns with previous research findings [5,21].

To provide a more comprehensive understanding, we integrated both quantitative and qualitative findings. Quantitative data showed that overall confidence levels and the number of correct answers increased when using Google. In some cases, confidence levels increased even when the answers were incorrect, possibly owing to extensive information examination [32]. Additionally, the number of participants who initially answered incorrectly but then correctly using Google search increased to 64 out of the 72 total questions. After answering each case vignette, the questionnaire results indicated that the difficulty level of the questions matched the preset level and that 70.1% of participants were proficient in using Google search. As participants received a lecture on using Google search as an information retrieval tool rather than a structured CDSS, unfamiliarity with Google search had minimal impact on the diagnostic accuracy and processes. Qualitative analysis provided deeper insights into the reasoning process behind these trends. Google search likely contributed to problem representation in the group with increased confidence in the diagnostic process. Examination of the subcategories within this category suggests that the input is more significant and that the selection of output information is a factor in increasing confidence. This also indicates the possibility of avoiding bias by reducing assumptions. When Google search was used to generate information about a disease identified early, participants became more confident that the disease they recalled was correct. Conversely, “diagnostic justification” was most involved in the group with lower confidence, where the variety of diseases in search results caused confusion. This confusion was likely due to a lack of sufficient medical knowledge to filter the extensive information provided by Google effectively. Future studies should explore whether this effect persists among more experienced physicians.

The integration of these findings suggests that while Google search use generally improves confidence and diagnostic accuracy, it also introduces potential biases. Unlike traditional CDSSs, Google search does not structure its output based on patient data, making it more susceptible to automation bias and misinformation. For example, automation bias and the Dunning-Kruger effect [33] may explain why confidence increased even when diagnoses were incorrect. This highlights the need for training that emphasizes critical evaluation of Google output. The biggest disadvantage of using such systems is that medical students may believe in all the output content without critically analyzing its accuracy [34].

In the focus group interviews, some respondents commented that they would pay attention to information about the first disease they recalled and look for it. To address such issues, it is essential to incorporate training that emphasizes the limitations and potential risks of automation bias alongside traditional medical education to ensure future healthcare professionals can use these tools effectively. These strategies will encourage critical evaluation of CDSS output, fostering balanced decision making without undermining clinical judgment. By embedding these elements into medical curricula, we can better educate students to integrate technology with clinical expertise. At the same time, diagnostic errors caused by overconfidence in search engines can be avoided. While Google search provides vast amounts of information rapidly, it also presents a challenge in the form of information overload. Without the ability to critically evaluate the relevance and reliability of the provided information, the risk of diagnostic errors increases. Content analysis revealed that participants who focused on selecting and evaluating output information were able to reinforce their diagnostic confidence, whereas those who struggled with information overload experienced decreased confidence. Therefore, the ability of healthcare professionals, including medical students, to discern reliable information from unreliable sources is crucial in avoiding incorrect diagnoses. Indeed, the results of our content analysis allowed us to delve deeper into the nuances of this issue.

For example, the subcategories of “selecting and choosing output information” and “evaluation of output information” within the broader category of “problem expression” were particularly prominent in the group that reported increased confidence. This suggests that those who focused on evaluating and filtering the provided information were able to reinforce their diagnostic confidence. Conversely, the subcategory “confusion due to output information” within the “diagnostic justification” category was more commonly observed in participants who experienced decreased confidence. This confusion may be attributed to an inability to process and prioritize the output from Google search effectively, underscoring the necessity of adequate training in information evaluation. The potential for confusion highlights the need for medical curricula to incorporate targeted training on search engines and CDSSs, to ensure balanced and effective use. Developing critical analysis skills can help healthcare professionals integrate CDSSs into clinical decision-making while maintaining sound judgment. Structured training on assessing search engines and CDSS output, selecting relevant information, and recognizing cognitive biases may support students’ diagnostic accuracy and confidence. Additionally, simulation-based exercises and reflective learning strategies could help students refine their ability to use diagnostic support tools effectively. Implementing these measures in medical curricula may better prepare future healthcare professionals to utilize diagnostic support tools while maintaining critical thinking and independent clinical judgment.

A lack of medical knowledge can cause decreased diagnostic confidence, but it could also occur when an actual patient’s situation does not match the information obtained on diagnostic support tools well. The use of tools may also undermine the autonomy of physicians [35]. As the case vignettes used in this study were typical of high-frequency diseases, it is easy to imagine that decreased diagnostic confidence could occur in more complex cases or rare diseases, and validating those cases is necessary. We should explore whether similar improvements in diagnostic accuracy and confidence are observed when dealing with challenging or uncommon conditions. Investigating such cases may provide further insights into the role of diagnostic support tools in clinical reasoning and medical education. Furthermore, we must consider whether the output information applies to a patient in conjunction with the symptoms. Assessing the extent to which users are prepared to use diagnostic support tools is necessary [36]. As these systems are utilized, clarifying the beneficial and disadvantageous aspects of CDSS in medical education is expected to lead to efficient learning by focusing on what medical students need to learn. Mastering these tools is an essential skill for future healthcare professionals, and the supplementary use of diagnostic support tools is expected to help prevent diagnostic errors and oversight.

## Limitation

This study had some limitations. First, it was a single-institution design targeting medical students at this university, which limits the findings’ generalizability and may result in low external validity. All participating medical students passed Japan’s standardized examinations, which assured a minimum level of knowledge; however, standardization is difficult because it is possible that some highly capable students were included. Additionally, while all students had sufficient medical knowledge, their proficiency in using semantic qualifiers (SQ) for search queries may have varied. Since we did not assess their prior SQ training, differences in SQ usage ability could have influenced search effectiveness and diagnostic accuracy. Second, the gender distribution of participants was biased, with 89.5% being male. This gender imbalance reflects the demographic composition of the medical school cohort at Chiba University during the study period and is also common across many medical schools in Japan. Although previous research suggests that gender differences may influence diagnostic reasoning and confidence, our study did not specifically examine this aspect. Future research should investigate the potential impact of gender on the use of CDSSs and diagnostic accuracy. Third, because participants were medical students, it is unclear whether the findings could be applied to residents or experienced physicians. Fourth, the case vignettes used for evaluation were limited to ten high-frequency cases that should be learned by resident physicians, highlighting the need for validation with more complex cases and rare diseases. Fifth, the high-frequency cases were relatively straightforward, with specific symptoms from which input keywords could be easily extracted. The case vignettes were paper-based and described using medical terminology, making them easy to understand and search for patient symptoms. This differs from the complaints of patients who present with diverse symptoms in clinical settings. While we assessed the overall difficulty of the cases, we did not evaluate the difficulty level of each case individually. While the cases were based on the Japanese national medical licensing exam and pretested by physicians, there may have been some variability in difficulty. Sixth, this study found that the content of the outputs influenced the diagnostic process; however, it was not possible to evaluate or otherwise analyse the content of the outputs. Future research would include analysis of the cognitive process of medical diagnosis and the strategies used by the students to validate the outputs, depending on the results of the outputs. Finally, although this study used Google search, validating the evaluation using other systems is also necessary. Future studies should compare different CDSSs, including artificial intelligence-based systems such as ChatGPT. Evaluating these systems’ effectiveness in improving diagnostic accuracy and confidence would enhance the generalizability of our findings and provide deeper insights into the optimal integration of CDSSs in medical education.

## Conclusion

This study highlights the value of using Google search in medical education to enhance diagnostic skills and confidence. The improvement in diagnostic accuracy and confidence among medical students demonstrates the supportive role of Google search in clinical reasoning education. It emphasizes the necessity for educators to teach medical students how to analyze the output information from diagnostic support tools based on sufficient medical knowledge to ensure optimal aid medical education. By effectively utilizing such systems, students can appropriately evaluate vast amounts of medical information and efficiently conduct medical practice.

## Supporting information

S1 Samples Case vignette samples. (DOCX)

S1 Lists List of case vignettes. (DOCX)

S1 Table Interview guide. (DOCX)

S2 Table Working Definitions for the Different Components of Clinical Reasoning. (DOCX)

S1 Original Data Raw Data Confidence and Answer. (DOCX)

S2 Original Data Raw Data on Case Difficulty Ratings and Google Search Proficiency. (DOCX)

## Abbreviations

CDSS: clinical decision support systems
SQ: semantic qualifiers.
