# Supplementary material for: Relationship between diagnostic accuracy and self-confidence among medical students when using Google search: A mixed-method study
Source: PLoS One. 2025 Sep 19;20(9):e0332918. doi: 10.1371/journal.pone.0332918 (PMC12448958; doi:10.1371/journal.pone.0332918)
Supplement: S1 Samples — (DOCX) [file pone.0332918.s001.docx]

**S1 Samples. Case vignette samples**

Sample 1. Acute sinusitis

Two weeks prior to the appointment, a 36-year-old man described having nasal discharge, cough, and fever. Despite taking over-the-counter medications, his symptoms subsided for approximately only three days. He visited the clinic five days before the appointment because of purulent nasal discharge, nasal discharge that ran down his mouth, and soreness around his right cheek. There is no prior medical history for this patient.

Sample 2. Acute epiglottitis

The overall health of a three-year-old boy was considered acceptable, but he had a fever and sore throat for three days. He was unable to eat on the morning of the appointment because of a sore throat, and subsequently started to cough and slobber without swallowing. The patient’s mother then brought him to the clinic for further examination. There is no prior medical history for this patient.
