# Supplementary material for: Relationship between diagnostic accuracy and self-confidence among medical students when using Google search: A mixed-method study
Source: PLoS One. 2025 Sep 19;20(9):e0332918. doi: 10.1371/journal.pone.0332918 (PMC12448958; doi:10.1371/journal.pone.0332918)
Supplement: S1 Lists — (DOCX) [file pone.0332918.s002.docx]

**S1 Lists. List of case vignettes**

| Case | Diagnosis |
| --- | --- |
| 1 | Transient ischemic attack |
| 2 | Pyelonephritis |
| 3 | Acute epiglottitis |
| 4 | Panic disorder |
| 5 | Cholecystitis |
| 6 | Lumbar spinal stenosis |
| 7 | Pulmonary thromboembolism |
| 8 | Benign paroxysmal positional vertigo |
| 9 | Acute angle-closure glaucoma |
| 10 | Acute sinusitis |
