# Supplementary material for: Relationship between diagnostic accuracy and self-confidence among medical students when using Google search: A mixed-method study
Source: PLoS One. 2025 Sep 19;20(9):e0332918. doi: 10.1371/journal.pone.0332918 (PMC12448958; doi:10.1371/journal.pone.0332918)
Supplement: S1 Table — (DOCX) [file pone.0332918.s003.docx]

**S1 Table. Interview guide**

1. General briefing.
2. Interview content.

Q1: *What is the change in confidence after using the CDSS and why?*

Q2: *Do you think the use of CDSS will reduce or increase diagnostic errors (misdiagnosis) and why?*

1. Closing remark.
